# Culture of Oral Mucosal Epithelial Cells for the Purpose of Treating Limbal Stem Cell Deficiency

**DOI:** 10.3390/jfb7010005

**Published:** 2016-03-01

**Authors:** Tor Paaske Utheim, Øygunn Aass Utheim, Qalb-E-Saleem Khan, Amer Sehic

**Affiliations:** 1 Department of Medical Biochemistry, Oslo University Hospital, Oslo 0407, Norway; utheim2@gmail.com; 2 Department of Oral Biology, Faculty of Dentistry, University of Oslo, Oslo 0372, Norway; 3 Department of Ophthalmology, Drammen Hospital, Vestre Viken Hospital Trust, Drammen 3004, Norway; 4 Department of Ophthalmology, Oslo University Hospital, Oslo 0407, Norway; outheim@gmail.com; 5 Department of Medical Biology, Faculty of Health Sciences, University of Tromsø, Tromsø 9037, Norway; qalb-e-saleem.k.ahmed@uit.no

**Keywords:** cornea, limbal stem cell deficiency, ocular surface disease, oral mucosal epithelial cell sheet, substrates

## Abstract

The cornea is critical for normal vision as it allows allowing light transmission to the retina. The corneal epithelium is renewed by limbal epithelial cells (LEC), which are located in the periphery of the cornea, the limbus. Damage or disease involving LEC may lead to various clinical presentations of limbal stem cell deficiency (LSCD). Both severe pain and blindness may result. Transplantation of cultured autologous oral mucosal epithelial cell sheet (CAOMECS) represents the first use of a cultured non-limbal autologous cell type to treat this disease. Among non-limbal cell types, CAOMECS and conjunctival epithelial cells are the only laboratory cultured cell sources that have been explored in humans. Thus far, the expression of p63 is the only predictor of clinical outcome following transplantation to correct LSCD. The optimal culture method and substrate for CAOMECS is not established. The present review focuses on cell culture methods, with particular emphasis on substrates. Most culture protocols for CAOMECS used amniotic membrane as a substrate and included the xenogeneic components fetal bovine serum and murine 3T3 fibroblasts. However, it has been demonstrated that tissue-engineered epithelial cell sheet grafts can be successfully fabricated using temperature-responsive culture surfaces and autologous serum. In the studies using different substrates for culture of CAOMECS, the quantitative expression of p63 was generally poorly reported; thus, more research is warranted with quantification of phenotypic data. Further research is required to develop a culture system for CAOMECS that mimics the natural environment of oral/limbal/corneal epithelial cells without the need for undefined foreign materials such as serum and feeder cells.

## 1. Introduction

### 1.1. Limbal Stem Cell Deficiency

The regenerating organs in the body (e.g., cornea, skin, and gut) harbor tissue-specific stem cells, which are responsible for tissue homeostasis and efficient healing in case of injury. The ocular surface is composed of corneal and conjunctival epithelium [1]. The corneal epithelium in particular plays a crucial role in maintaining the cornea’s avascularity and transparency [2]. The self-renewal of the corneal surface is a multistep process dependent on a small population of limbal stem cells [3,4] located in structures referred to as limbal crypts [5] or limbal epithelial crypts [6].

Numerous external factors and disorders (e.g., chemical or thermal injuries, microbial infections, surgeries involving the limbus, cicatricial pemphigoid, and aniridia) can lead to dysfunction or loss of limbal epithelial cells (LEC), resulting in either partial or total limbal stem cell deficiency (LSCD) [2]. The condition can be painful and may lead to reduced vision, or even blindness, by causing persistent epithelial defects, fibrovascular pannus, conjunctivalization, and superficial and deep vascularization of the cornea. The persistence of epithelial defects may result in ulceration, scarring, and corneal perforation [2]. Limbal stem cell deficiency is most often bilateral.

### 1.2. Treatment Strategies for Limbal Stem Cell Deficiency

Treatment approaches for LSCD can be categorized as follows: (a) transplantation of cultured cells [2]; (b) transplantation of non-cultured cells [2]; and (c) approaches that do not involve transplantation of cells, for example keratoprostheses [7]. A great variety of cell-based therapeutic strategies have been suggested for LSCD [8]. The stem cells of the corneal epithelium are believed to be located in the limbus [3,4]. In 1989, limbal grafts were transplanted to eyes suffering from LSCD to restore the corneal surface [9]. The results were promising. However, the procedure carries a risk of inducing LSCD in the healthy eye because of large limbal cell withdrawal [10], and the therapy is not possible in cases of bilateral LSCD. This led to a novel therapeutic strategy with *ex vivo* expansion of LEC first reported by Pellegrini and colleagues in 1997 [11]. In their study, successful ocular surface reconstruction was achieved using autologous cultivated LEC isolated from small biopsies in two patients, both affected with severe unilateral ocular surface disease. Since then, more than 1000 patients suffering from LSCD have been treated with *ex vivo* cultured LEC [11,12,13,14,15,16,17,18]. Since 2003, nine cultured non-limbal cell sources have been successfully used to reconstruct the corneal epithelium in bilateral LSCD, in which limbal tissue is not recommended for harvest [8]. The sources include oral mucosal epithelial cells [19], embryonic stem cells [20], conjunctival epithelial cells [21], epidermal stem cells [22], dental pulp stem cells [23], bone marrow-derived mesenchymal stem cells [24], hair follicle bulge-derived stem cells [25], umbilical cord lining stem cells [26], and orbital fat-derived stem cells [27]. Among non-limbal cell types, cultured autologous oral mucosal epithelial cell sheet (CAOMECS) and conjunctival epithelial cells are the only laboratory cultured cell sources that have been explored in humans.

## 2. Cultured Autologous Oral Mucosal Epithelial Cell Sheet

A significant advantage of CAOMECS as a cell source is easy isolation from biopsies that heal quickly without residual scarring. As the CAOMECS are autologous, there is no risk of immune rejection, thus making immunosuppression unnecessary. However, a disadvantage of transplantation of CAOMECS is the development of peripheral neovascularization [28,29,30,31]. Studies have demonstrated that angiogenesis related factors were expressed in corneas after transplantation [32,33,34,35]. Anti-angiogenic therapy has been proposed as a method to prevent corneal neovascularization and improve the outcomes after transplantation with CAOMECS [36]. Thus far, 242 patients with LSCD have been reported as treated, with a success rate of 72% and a follow-up time of between one and 7.5 years [19,28,29,30,31,32,37,38,39,40,41,42,43,44,45,46,47,48,49,50,51].

An ideal substrate is easily available, transparent, and easy to manipulate; it permits cells to proliferate and retain high viability. Though transplant success has been demonstrated using various culture methods, the optimal culture method for CAOMECS for use in corneal regeneration has not been established. The determination of appropriate substrates and culture protocols for CAOMECS may contribute to the development of standardized, safe, and effective regenerative therapy for LSCD. The present review focuses on the current state of knowledge of the culture methods and substrates used for CAOMECS in ocular regeneration. The review was prepared by searching the National Library of Medicine database using the search term “oral mucosal epithelial cells” in an attempt not to leave out any relevant publications. In total, the search resulted in 4897 studies, of which 41 studies, published from 2003 to 2015, were related directly to the core topic of the present review.

## 3. Characteristics of the Culture Protocol for Cultured Autologous Oral Mucosal Epithelial Cell Sheet

The standard culture conditions used for production of transplantable epithelial cell sheets, including CAOMECS, typically requires fetal bovine serum (FBS) and murine 3T3 feeder layers [52]. The epithelial progenitor or stem cells isolated from small biopsies can, under these conditions, be expanded *in vitro* to create stratified epithelial layers that closely resemble native tissues [53]. However, these constructs are classified as xenogeneic products, with the inherent possibility of infection or pathogen transmission from animal-derived materials [54]. In addition, xeno-contamination may result in immunogenicity [55]. The use of feeder layers and foreign serum is, therefore, a concern in regenerative medicine. Furthermore, dispase, a bacteria-derived protease, is commonly used to enable cell isolation [53].

**Figure 1:**
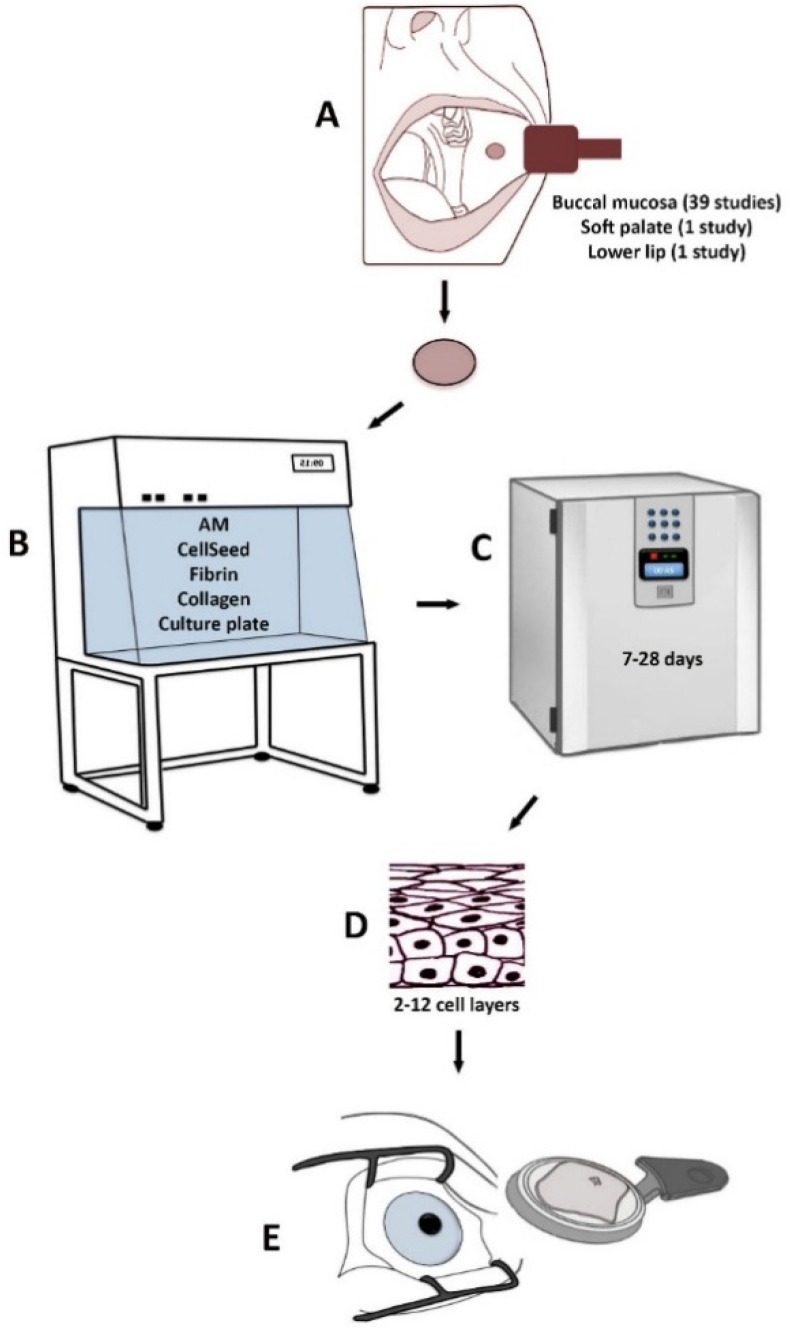
Treatment of LSCD based on various methods using CAOMECS. A biopsy from the mucosa is harvested from the oral cavity (**A**). The biopsy is cultured in the laboratory on different substrates (**B**) for 7–28 days (**C**). A stratified cultured tissue is produced (**D**) and is transplanted to the diseased eye (**E**).

Treatment of LSCD based on various methods using CAOMECS is presented in Figure 1. The following culture methods and substrates have been used in order to produce transplantable CAOMECS: (1) amniotic membrane [28,29,30,32,35,37,39,40,42,43,45,46,47,49,51,56,57,58,59,60,61,62,63] (Table 1); (2) temperature-responsive cell-culture surfaces [31,38,64,65,66,67,68,69,70] (Table 2); (3) fibrin-coated culture plates [41,48] (Table 3); (4) fibrin gel [71] (Table 3); (5) collagen IV-coated culture plates [72] (Table 3); and (6) culture plates without any substrate [33,34,73,74] (Table 3).

The possibility of pathogen transmission cannot be excluded from xenogeneic or allogeneic materials, such as human amniotic membrane obtained following elective Caesarean operations [17,18,75], collagen isolated from porcine or bovine skin [76], and hydrated gels made from fibrin derived from human donor blood [77,78,79]. Therefore, the establishment of culture conditions avoiding animal-derived products and foreign undefined components is warranted.

## 4. Culture of Oral Mucosal Epithelial Cells on Amniotic Membrane

Amniotic membrane has been used on the ocular surface since 1940 [80], and for the first time in treatment of LSCD in 1946 [81]. In cases of partial LSCD, amniotic membrane can be applied to the affected eye and provide a suitable substrate for corneal epithelial repopulation [82,83]. The amniotic membrane secretes several growth factors such as hepatocyte growth factor, basic fibroblast growth factor, and transforming growth factor β [84,85]. Amniotic membrane is suggested to exert its effects by suppressing inflammation and scarring [86]. There is currently a discussion over whether amniotic membrane should be deepithelialized/denuded prior to culture, or if this substrate should remain intact. It has been reported that native, intact amniotic membrane comprise higher levels of growth factors compared to denuded amniotic membrane [87].

Amniotic membrane is the most common culture substrate for CAOMECS, and has been used in 15 clinical, three animal, and six *in vitro* studies (Table 1). With one exception [43], the amniotic membrane was denuded, *i.e.*, the single layer of epithelial cells on the amniotic membrane was removed (Table 1). In the studies using amniotic membrane as a substrate for cultured CAOMECS, cell suspension [28,29,30,32,35,37,39,42,45,46,47,49,50,51,56,57,58,61,62,63] was applied in all studies, except four using the explant method [40,43,59,60]. The number of fabricated, stratified epithelial cell layers varied from two [56] to 10 [32]. Oral mucosal epithelial cells were normally cultivated between two to three weeks; however, the culture time varied between seven [62] and 28 [63] days. The most frequently used culture medium with added supplements was Dulbecco`s Modified Eagle Medium (DMEM:F12) [28,29,30,35,39,40,42,43,46,47,49,50,51,56,57,58,59,60,61,63], followed by keratinocyte growth medium (KGM) [37,45] and supplemented hormonal epithelial medium (SHEM) [32,62] (Table 1). Murine fibroblasts (3T3 strain) were used in all but three studies [40,43,56]. Most of the culture protocols exposed the cells to air-lifting (lowering the level of the culture medium to allow the cells to be cultured at the air–liquid interface), including clinical [28,30,37,42,43,45,47,49,50,51], animal [57,58], and *in vitro* studies [35,59,60,63] (Table 1). Fetal bovine serum (FBS) [29,30,37,47,57,58,59,61,62,63] and fetal calf serum (FCS) [28,32,39,42,56,60] were broadly used; however, six studies used human autologous serum (HAS) [28,43,45,46,49,50] in an attempt to minimize/avoid the use of animal derived components (Table 1).

Oral mucosal epithelial cells cultivated on amniotic membrane exhibited multilayered, stratified epithelium and appeared very similar to a normal corneal epithelium (Table 1). The presence of non-keratinized, stratified-specific keratins K3 and K4/K13 was detected by immunohistochemistry, reverse transcription polymerase chain reaction, and Western blotting (Table 1). The expression of p63, a marker for undifferentiated cells, was reported in 33% (8/24) of the studies (Table 4). Using transmission electron microscopy it was demonstrated that the cultivated oral epithelial sheet had junctional contacts, such as desmosomes, hemidesmosomes, and tight junctions, which were almost identical to those of normal corneal epithelial cells [30,56,60,62].

## 5. Culture of Oral Mucosal Epithelial Cells on Temperature- Responsive Surfaces

In order to avoid the use of allogenic bacteria [53,67] and animal derived [52] components in the cornea-engineered constructs, carrier-free epithelial cell sheets using temperature-responsive culture dishes have been developed [31,88,89]. The modified surfaces transition between hydrophilic and hydrophobic states—depending on the temperature—by covalently immobilizing the temperature-responsive polymer poly(*N-*isopropylacrylamide) onto commercially available tissue culture wells. Under *in vitro* culture conditions at 37 °C, numerous cell types adhere and proliferate similarly to those of normal tissue culture polystyrene. By reducing the temperature to 20 °C, the cultured cells spontaneously detach along with their deposited extracellular matrix (ECM) without the need for proteolytic enzymes such as dispase [89,90]. Therefore, with temperature-responsive culture surfaces the undesirable factors inherent to some substrates can be excluded from transplantable constructs.

Nine studies (three clinical, four animal, and two *in vitro*) have utilized the temperature-responsive cell-culture surfaces as a substrate for CAOMECS. In all studies the cells were applied as a cell suspension and DMEM:F12 with added supplements was used as a culture medium (Table 2). The culture time for CAOMECS in these studies ranged from 10 [69] to 28 [65] days, but was most often two weeks [31,64,66,67,70]. The most common nutrient used was FBS [64,65,66,69,70]; however, two studies utilized HAS [67,68]. None of the studies exposed the cells to air-lifting (Table 2). The number of fabricated cell layers varied from three [69] to eight [65]. Only one study did not use 3T3 murine fibroblasts [67]. Two studies reported the cell viability of the cultured sheets to be 86% [68] and 93% [65]. The presence of p63 in the fabricated cell sheets was reported in 78% (7/9) of the studies (Table 4).

## 6. Culture of Oral Mucosal Epithelial Cells on Fibrin Substrates

Fibrin has been broadly used as a substrate in regenerative medicine and for wound-healing [91,92]. It is easily available, assists epithelial cell growth, and its degradation can be controlled by addition of fibrinolytic components. Rama and colleagues first established the use of fibrin gels as a substrate for ocular surface reconstruction in 2001 [78]. Fibrin gel is a hemostatic compound of thrombin, fibrinogen, and calcium chloride [93]. The mixture of these components fabricates a gel that is similar to the physiological lump formed at the last stage of the coagulation cascade [94]. The gel produced by this reaction is biodegradable, non-toxic, and inhibits fibrosis, tissue necrosis, and inflammation [94,95,96]. *In vivo*, the gel is completely resorbed and ultimately replaced by matrix components such as collagen [95]. A major disadvantage with fibrin as a substrate is that it encourages angiogenesis [97]. The gel, however, is resorbed within days to weeks after transplantation [94], minimizing the effects. Sheth *et al.* have demonstrated that CAOMECS cultivated on fibrin gel results in production of multilayered epithelium *in vitro*. The fabricated cell sheets expressed keratins K3, K4, and K13 [71]. The putative epithelial progenitor cell marker p63 [98] was also highly expressed (Table 3). Sheth and associates modified the pre-existing methodology to produce a reproducible, robust gel that supports the expansion and transplantation of CAOMECS, without the need for murine 3T3 fibroblasts. Fibrin-coated culture plates have also been used as a substrate for CAOMECS [41,48] (Table 3). Both studies utilized murine 3T3 fibroblasts and DMEM:F12 with added supplements as a culture medium. Human autologous serum was used as nutrient, and the cells were exposed to air-lifting [41,48].

## 7. Culture of Oral Mucosal Epithelial Cells on Collagen Substrates

All of the previously reported culture protocols for CAOMECS use serum, and most also use feeder cells to support the stratification of the epithelial cells. Due to the risk of infections associated with murine feeder cells and non-autologous serum in the cultivation of cell sheets, Ilmarinen and colleagues sought other options to support the stratification of isolated CAOMECS [72]. In their *in vitro* study, stratified epithelium was generated on collagen IV-coated culture plates in serum-free culture conditions without using 3T3 feeder cells. The authors analyzed the functional properties of the cell sheets by transepithelial electrical resistance measurements, in addition to morphology, differentiation, and regenerative capacity. This study is the only report of a successful stratification of oral mucosal epithelium for ocular surface regeneration in the absence of serum. The results showed that, in serum-free conditions, oral mucosal epithelial cells attached to and proliferated on collagen IV–coated inserts more readily than on amniotic membrane [72]. Ilmarinen and colleagues also studied the effects of increased epidermal growth factor (EGF) concentration, as EGF is known to stimulate the growth and differentiation of a variety of epithelial tissues [99,100]. However, they detected no major effects on the phenotype of the cell sheets using additional EGF.

## 8. Culture of Oral Mucosal Epithelial Cells on Non-Coated Culture Plates

Four studies (three *in vitro* and one animal) have used non-coated, substrate-free culture plates in order to fabricate transplantable CAOMECS [33,34,73,74] (Table 3). All of the studies used DMEM:F12 with added supplements as a culture medium and FBS as a nutrient, without including air-lifting. In three studies, murine 3T3 feeder cells were included [33,34,74]. The authors reported formation of a multilayered epithelium [33,34,73], one study specifying the number of cell layers [74]. Two of the four studies confirmed the expression of K3 and high expression of p63 [73,74].

## 9. Challenges and Future Perspectives

Recently, a meta-analytic concise review about transplantation of CAOMECS for treating LSCD has reported a success rate of 72% [19]. In this review, the focus was on clinical features of transplants of CAOMECS over the past 10 years, including surgery and pre- and postoperative considerations. In contrast, herein we focus on cell culture methods, with particular emphasis on substrates. Moreover, in the present review we expand on both *in vitro* and animal studies. 

A complete xenobiotic-free culture protocol has become a goal in regenerative medicine; this is to avoid the risk of transferring known and unknown microorganisms and to standardize the culture conditions. The properties of epithelial cells are dependent upon extracellular signals supplied by the cell–cell and cell–substratum interactions. Further research is warranted to develop a culture system for CAOMECS that mimics the natural environment of oral/limbal/corneal epithelial cells without the need for undefined foreign materials such as serum and feeder cells.

It is likely that the phenotype of CAOMECS affects clinical success following transplantation. Thus far, p63 is the only predictor of clinical outcome following transplantation to correct LSCD [12]. Recently, Rama *et al.* demonstrated that the phenotype of cultured LEC is critical to ensure successful reconstruction of the ocular surface following LSCD [12]. The authors showed that successful transplantation was achieved in 78% of patients when using cell cultures in which p63-bright cells constituted more than 3% of the total number of clonogenic cells. In contrast, successful transplantation was only seen in 11% of patients when p63-bright cells made up 3% or less of the total number of cells. In the studies using different substrates for culture of CAOMECS, the expression of p63 varied considerably (Table 4). Few studies reported the expression of p63 when using fibrin-coated culture plates, fibrin gels, collagen-coated culture plates, and culture plates without substrate (Table 4). When comparing amniotic membrane and temperature-responsive inserts, 33% (8/24) and 78% (7/9) of the studies showed the expression of p63, respectively (Table 4). The quantitative expression of p63 was generally poorly reported; thus, more research is warranted with quantification of phenotypic data.

The use of culture inserts with autologous serum has also been shown to facilitate the stratification of oral mucosal epithelial cells in the absence of 3T3 feeders [67]. Kolli *et al.* found that autologous serum was superior to FCS in generating an undifferentiated epithelium [43], and in another study the porcine trypsin was replaced with xeno-free trypsin with successful outcomes [61]. Hirayama *et al.* [41] showed that transplantation of a substrate-free cell sheet resulted in significantly better results than engrafting oral mucosal cells cultured on an amniotic membrane. The improvements were significantly higher graft survival rate, better visual acuity (1, 3, 6, and 12 months postoperatively), and reduction of neovascularization (12 months postoperatively) [41]. Furthermore, except collagen IV-coated culture plate, this review demonstrates that the use of different methods and substrates for culture of CAOMECS did not appear to have any effect on the number of cell layers generated (Table 5).

Due to the lack of mechanical strength provided by various culture substrates, transplantation of substrate-free cell sheets can be challenging. Hence, methods to enhance the strength and durability of the epithelial cell sheets should be further explored. Using the air-lifting technique, originally developed to formulate skin cell culture sheets for transplantation, the mechanical strength of epithelial cell sheets can be increased. The present review reveals that only 48.8% of the studies applied the air-lifting method (Table 1, Table 2 and Table 3). Interestingly, the majority of studies using amniotic membrane (71%) did utilize air-lifting, while none of the studies with temperature-responsive surfaces applied this method (Table 5). Arguments for air-lifting include the promotion of migration [101], proliferation [101], epithelial stratification [101], and increased barrier function of LEC [102]. Arguments against air-lifting include induction of squamous metaplasia [103], gradual loss of stem cells [104], and differentiation of LEC [104,105]. Until 2010, the clinical implications of increased differentiation of transplanted cells in corneal reconstruction were unknown. This changed when Rama and colleagues demonstrated the critical importance for clinical success of a substantial, putative stem cell population within the cultured cells [12]. It is yet to be investigated whether the potential advantages of air-lifting outweigh the disadvantages in corneal regeneration using CAOMECS.

## 10. Conclusions

Most culture protocols for CAOMECS used amniotic membrane as a substrate and included the xenogeneic components FBS and murine 3T3 fibroblasts. However, it has been demonstrated that tissue-engineered epithelial cell sheet grafts can be successfully fabricated using temperature-responsive culture surfaces and autologous serum. More studies on how various substrates and other culture parameters affect the cell sheet, with special emphasis on the phenotype, are warranted. Furthermore, it is important to focus on cell culture methods using xenobiotic-free conditions.

## Figures and Tables

**Table 1:** Culture of oral mucosal epithelial cells on amniotic membrane.

Author, Year	Type of Study	Cell Suspension/Explant	Substrate	Air-Lifting	Serum	3T3	Culture Medium	Culture Time (Days)	Morphology	Phenotype
Shimazaki *et al.*, 2009 [62]	Animal	Cell suspension	Denuded AM	Yes	FBS	Yes	SHEM (aprotinin)	7–10	Multilayered stratified epithelium; Tight junctions	Expression of K3, ZO-1, and occludin
Sekiyama *et al.*, 2006 [35]	*in Vitro*	Cell suspension	Denuded AM	Yes	–	Yes	DMEM:F12 (penicillin, streptomycin, insulin, cholera toxin, EGF)	7–14	–	Expression of VEGF and Flt-1; Low expression of PEDF
Sotozono *et al.*, 2013 [50]	Clinical	Cell suspension	Denuded AM	Yes	HAS	Yes	DMEM:F12 (penicillin, streptomycin, insulin, cholera toxin, EGF)	8–9	–	–
Sotozono *et al.*, 2014 [49]	Clinical	Cell suspension	Denuded AM	Yes	HAS	Yes	DMEM:F12 (penicillin, streptomycin, insulin, cholera toxin, EGF)	8–9	–	–
Gaddipati *et al.*, 2014 [40]	Clinical	Explant	Denuded AM	–	–	No	DMEM:F12 (penicillin, streptomycin, insulin, cholera toxin, EGF)	9	5–6 cell layers; Stratified epithelium	Expression of K3, K12, K19, Ki-67, p75, and PAX6; p63 expression in most of the basal and supra basal cells
Sen *et al.*, 2011 [60]	*in Vitro*	Explant	Denuded AM	Yes	FCS	Yes	DMEM:F12 (penicillin, streptomycin, amphotericin, EGF, insulin)	14	Stratified epithelium; Desmosomes; Abundant mucin granules	Expression of K3, K4, K13, connexin 43, p63, p75, β_1_-integrin, CD29, ABCG2, and MUC 1, 5B, 6, 13, 15 and 16
Satake *et al.*, 2008 [47]	Clinical	Cell suspension	Denuded AM	Yes	FBS	Yes	DMEM:F12 (gentamycin, streptomycin, penicillin, amphotericin, EGF, insulin)	>14	Non-keratinized, squamous, polygonal, cells with a low nuclear to cytoplasmatic ratio	–
Takeda *et al.*, 2011 [51]	Clinical	Cell suspension	Denuded AM	Yes	–	Yes	DMEM:F12 (penicillin, streptomycin, insulin, cholera toxin, EGF)	14–16	–	–
Chen *et al.*, 2009 [39]	Clinical	Cell suspension	Denuded AM	No	FCS	Yes	DMEM:F12(penicillin, streptomycin, insulin, cholera toxin, EGF)	14–21	2–5 cell layers; Elongated cell nuclei	Expression of K3, K4, K13, p63, p75, and ABCG2
Chen *et al.*, 2012 [32]	Clinical	Cell suspension	Denuded AM	No	FCS	Yes	SHEM (penicillin, streptomycin, insulin, cholera toxin, EGF)	14–21	5–10 cell layers; Stratified epithelium	Expression of FGF2, K8, VEGF, endostatin, PEDF, and IL-1ra
Ma *et al.*, 2009 [29]	Clinical	Suspension	Denuded AM	No	FBS	Yes	DMEM:F12 (penicillin, streptomycin, insulin, cholera toxin, EGF)	14–21	2–5 cell layers; Elongated cell nuclei	Expression of K3, K13, p63, p75, and ABCG2
Nakamura *et al.*, 2004 [30]	Clinical	Cell suspension	Denuded AM	Yes	FBS	Yes	DMEM:F12 (penicillin, streptomycin, insulin, cholera toxin, EGF)	14–21	5–6 cell layers; Desmosomes and hemidesmosomes	Expression of K3, K4, and K13
Ang *et al.*, 2006 [37]	Clinical	Cell suspension	Denuded AM	Yes	HAS/FBS	Yes	KGM (penicillin, streptomycin, insulin, EGF)	15–16	4–6 cell layers; Cuboidal cells, More flattened cells superficially	Expression of K3, K4, K13, ZO-1, desmoplakin, integrin-α_1_, laminin 5, and collagen IV
Ang *et al.*, 2006 [37]	Clinical	Cell suspension	Denuded AM	Yes	HAS/FBS	Yes	KGM (penicillin, streptomycin, insulin, EGF)	15–16	4–6 cell layers; Cuboidal cells, More flattened cells superficially	Expression of K3, K4, K13, ZO-1, desmoplakin, integrin-α_1_, laminin 5, and collagen IV
Inatomi *et al.*, 2006 [28]	Clinical	Cell suspension	Denuded AM	Yes	FCS	Yes	DMEM:F12 (penicillin, streptomycin, insulin, cholera toxin, EGF)	15–16	5–6 cell layers; Cuboidal cells, several suprabasal cell layers, and flat apical cell layers	Expression of VEGF, FGF, and thrombospondin 1
Inatomi *et al.*, 2006 [28]	Clinical	Cell suspension	Denuded AM	Yes	FCS	Yes	DMEM:F12 (penicillin, streptomycin, insulin, cholera toxin, EGF)	15–16	5–6 cell layers; Cuboidal cells, several suprabasal cell layers, and flat apical cell layers	Expression of VEGF, FGF, and thrombospondin 1
Inatomi *et al.*, 2006 [42]	Clinical	Cell suspension	Denuded AM	Yes	HAS/FCS	Yes	DMEM:F12 (penicillin, streptomycin, insulin, cholera toxin, EGF)	15–16	5–6 cell layers; Cuboidal cells, several suprabasal cell layers, and flat apical cell layers	
Nakamura *et al.*, 2011 [45]	Clinical	Cell suspension	Denuded AM	Yes	HAS	Yes	KGM (penicillin, streptomycin, insulin, cholera toxin, EGF)	15–16	–	–
Priya *et al.*, 2011 [46]	Clinical	Cell suspension	Denuded AM	No	AS	Yes	DMEM:F12 (PI, mouse IgG1/IgG2a, mitomycin C, EGF, insulin, penicillin, streptomycin)	18–21	Flat and uniformly distributed epithelial cells	Low expression of p63 (3.0% ± 1.7% of cells); Negative expression of K12
Sharma *et al.*, 2011 [61]	*In vitro*	Cell suspension	Denuded AM	–	FBS	Yes	DMEM:F12 (penicillin, streptomycin, insulin, cholera toxin, EGF)	21	3–5 cell layers; Stratified epithelium	Expression of K3 and β_1_-integrin; High expression of p63
Promprasit *et al.*, 2014 [59]	*in Vitro*	Explant	Denuded AM	Yes	FBS	Yes	DMEM:F12 (penicillin, streptomycin, insulin, EGF)	21	2–5 cell layers; Stratified epithelium; Cuboidal cells in basal layer, flat superficial cells	Expression of K3 and connexin 43; High expression of p63
Nakamura *et al.*, 2003 [57]	Animal	Cell suspension	Denuded AM	Yes	FBS	Yes	DMEM:F12 (penicillin, streptomycin, insulin, cholera toxin, EGF)	21	3–5 cell layers; Stratified epithelium;	Expression of K3, K4, and K13
Nakamura *et al.*, 2003 [58]	Animal	Cell suspension	Denuded AM	Yes	FBS	Yes	DMEM:F12 (penicillin, streptomycin, insulin, cholera toxin, EGF)	21	5–6 cell layers; Stratified epithelium;	Expression of K3, K4, and K13
Nakamura *et al.*, 2003 [58]	Animal	Cell suspension	Denuded AM	Yes	FBS	Yes	DMEM:F12 (penicillin, streptomycin, insulin, cholera toxin, EGF)	21	5–6 cell layers; Stratified epithelium;	Expression of K3, K4, and K13
Kolli *et al.*, 2014 [43]	Clinical	Explant	Intact AM	Yes	HAS	No	DMEM:F12 (penicillin, streptomycin, insulin, cholera toxin, EGF, hydrocortisone, triiodothyronine, adenine)	21	3–7 cell layers, firmly attached to each other; High nucleus to cytoplasm ratio	Expression of K3, ABCG2, and C/EBPδ; High expression of ΔNp63α; Negative for K12 and PAX6
Madhira *et al.*, 2008 [56]	*in Vitro*	Cell suspension	Denuded AM	No	FCS	No	DMEM:F12 (penicillin, streptomycin, amphotericin, gentamycin, insulin, cholera toxin, EGF)	21–28	2–3 cell layers; Stratified epithelium; Gap junctions and desmosomes	Expression of K3, K4, K15, and connexin 43; Negative for K12 and PAX6
Yokoo *et al.*, 2006 [63]	*in Vitro*	Cell suspension	Denuded AM	Yes	FBS	Yes	DMEM/F12 (penicillin, streptomycin, amphotericin)	28	3–5 cell layers; Stratified epithelium	–

ABCG2, ATP binding cassette subfamily G member; AM, amniotic membrane; AS, autologous serum; DMEM, Dulbecco’s modified eagle medium; EGF, epidermal growth factor; FBS, fetal bovine serum; FCS, fetal calf serum; FGF2, fibroblast growth factor 2; Flt-1, Fms-like tyrosine kinase 1; HAS, human autologous serum; IgG2a, immunoglobulin G2a; IL-1ra, interleukin 1ra; KGM, keratinocyte growth medium; MUC, mucin; PAX6, paired box 6; PEDF, pigment epithelium derived factor; PI, propidium iodide; SHEM, supplemented hormonal epithelial medium; VEGF, vascular endothelial growth factor; ZO-1, zona occludens protein 1; –, indicates not reported.

**Table 2:** Culture of oral mucosal epithelial cells on temperature-responsive surfaces.

Author, Year	Type of Study	Cell Suspension/Explant	Substrate	Air-Lifting	Serum	3T3	Culture Medium	CultureTime (Days)	Morphology	Phenotype
Burillon *et al.*, 2012 [38]	Clinical	Cell suspension	CellSeed ^a^	No	–	Yes	–	–	Similar characteristics to normal corneal epithelium; Basal membrane	Expression of K3/76, p63, laminin 5, and β_1_-integrin
Soma *et al.*, 2014 [69]	Animal	Cell suspension	CellSeed ^a^	–	FBS	Yes	DMEM:F12 (insulin, triiodthyronine, hydrocortisone)	10–12	3–4 cell layers; Stratified epithelium; Cobble stone-like cell morphology	Expression of K14 and p63
Sugiyama *et al.*, 2014 [70]	Animal	Cell suspension	CellSeed ^a^	–	FBS	Yes	DMEM:F12 (penicillin, streptomycin, insulin, cholera toxin, EGF, hydrocortisone, triiodothyronine)	14	3–5 cell layers; Stratified epithelium; Cuboidal cells in the basal layer, squamous epithelium on the apical side	Expression of K4, K13, MUC5
Nishida *et al.*, 2004 [31]	Clinical	Cell suspension	CellSeed ^a^	No	–	Yes	–	14	Multilayered cell sheets; Microvilli, desmosomes, basement membrane	Expression of β_1_-integrin, K3, and p63
Bardag-Gorce *et al.*, 2015 [64]	Animal	Cell suspension	CellSeed ^a^	–	FBS	Yes	–	14	Multilayered stratified epithelium	Expression of K4, ΔNp63, TIMP-1, TIMP-3, and connexin 43
Hayashida *et al.*, 2005 [66]	Animal	Cell suspension	CellSeed ^a^	–	FBS	Yes	–	14	3–5 cell layers; Stratified epithelium;	Expression of K3, K4, K13, p63, ΔNp63, and β_1_-integrin
Murakami *et al.*, 2006 [67]	*in Vitro*	Cell suspension	CellSeed ^a^	–	HAS	No	DMEM/F12 (penicillin, streptomycin, fungizone, transferrin, EGF, cholera toxin, hydrocortisone, triiodothyronine)	14	3–5 cell layers; Cuboidal basal cells, flattened middle cells, and polygonal flattened superficial cells	Expression of p63 and Ki67
Oie *et al.*, 2010 [68]	Clinical	Cell suspension	CellSeed ^a^	–	HAS	Yes	–	14–17	4–5 cell layers; Small basal cells, flattened middle cells, and polygonal flattened superficial cells	Expression of K1, K3/76, K4, K10, K12, K13, K15, ZO-1, and MUC16; Moderate expression of p63 (30.7% ± 7.6% of cells)

**Table 3:** Culture of oral mucosal epithelial cells on other substrates.

Author, Year	Type of Study	Cell Suspension/Explant	Substrate	Air-Lifting	Serum	3T3	Culture Medium	Culture Time (Days)	Morphology	Phenotype
Satake *et al.*, 2011 [48]	Clinical	Cell suspension	Fibrin-coated cell culture inserts	Yes	HAS	Yes	DMEM:F12 (penicillin, streptomycin, transferrin, EGF, hydrocortisone, triiodothyronine)	–	5–6 cell layers;	–
Hirayama *et al.*, 2012 [41]	Clinical	Cell suspension	Fibrin-coated cell culture inserts	Yes	HAS	Yes	DMEM:F12 (penicillin, streptomycin, insulin, EGF, hydrocortisone)	–	5–6 cell layers;	–
Sheth *et al.*, 2014 [71]	*in Vitro*	Explant	Fibrin gel	–	FCS	No	DMEM:F12 (penicillin, cholera toxin, insulin, EGF, hydrocortisone)	-	Multilayered epithelium; Cobblestone morphology	Expression of K3, K13, and K19; High expression of p63
Ilmarinen *et al.*, 2012 [72]	*in Vitro*	Cell suspension	Collagen IV-coated cell culture inserts	Yes	No	No	Serum-free oral PCT epithelium medium (EGF)	13–17	4–12 cell layers; Stratified epithelium; Cuboidal basal cells and flat intermediate and superficial cells	Expression of K3/12, K4, K13, Ki67, and p63
Kanayama *et al.*, 2007 [34]	*in Vitro*	Cell suspension	Culture plate	–	FBS	Yes	DMEM (Supplements not reported)	–	Multilayered cells; Normal epithelial morphology	Expression of FGF2, VEGF, Ang1, and TGF-β1
Kanayama *et al.*, 2009 [33]	*in Vitro*	Cell suspension	Culture plate	–	FBS	Yes	DMEM (Supplements not reported)	14	Multilayered cells	Expression of VEGFr-1
Hyun *et al.*, 2014 [74]	Animal	Cell suspension	Culture plate	–	FBS	Yes	DMEM:F12 (penicillin, streptomycin, gentamycin, amphotericin)	14	2–6 cell layers; Stratified epithelium	Expression of K3, K4, and Ki67; High expression of p63
Krishnan *et al.*, 2010 [73]	*in Vitro*	Explant	Culture plate	–	FBS	–	DMEM:F12 (streptomycin, amphotericin, EGF, insulin, transferrin, selenium, hydrocortisone)	21	Multilayered cells; Normal epithelial morphology	Expression of ABCG2, K3, MUC1/4/16, hBD1/2,3; High expression of p63 and ΔNp63

ABCG2, ATP binding cassette subfamily G member; Ang1, angiopoietin; DMEM, Dulbecco’s modified eagle medium; EGF, epidermal growth factor; FBS, fetal bovine serum; FCS, fetal calf serum; FGF2, fibroblast growth factor 2; HAS, human autologous serum; hBD, human beta defensing; MUC, mucin; PCT, progenitor cell-targeted; TGF-β1, transforming growth factor beta 1; VEGF, vascular endothelial growth factor; –, indicates not reported.

**Table 4:** Expression of p63 in cultured autologous oral mucosal epithelial cell sheet cultivated on different substrates.

Substrate	Total Number of Studies	Expression of p63 Not Reported	Non-Quantitative Expression of p63 Reported	Quantitative Expression of p63 Reported
Amniotic membrane	24	16 studies	4 studies: p63 expressed; 1 study: high expression of ΔNp63; 2 studies: high expression of p63	1 study: 3.0% ± 1.7% of cells
Temperature-responsivecell-culture inserts	9	2 studies	6 studies: p63 expressed	1 study: 30.7% ± 7.6% of cells
Fibrin-coated culture plate	2	2 studies	–	–
Fibrin gel	1	–	1 study: high expression of p63	–
Collagen IV-coated culture plate	1	–	1 study: p63 expressed	–
Culture plate	4	2 studies	2 studies: high expression of p63	–

**Table 5:** Overall Effect of Different Culture Methods and Substrates for Cultured Autologous Oral Mucosal Epithelial Cell Sheet.

Substrate/Method	Air-lifting	Animal-derived Nutrient	Use of 3T3	Serum-free Medium	Viability	Morphology	Phenotype (Expression of p63)
Amniotic membrane	17/24	16/24	21/24	0/24	>98% (1)	4.2 cell layers (15)	++
Temperature-responsive cell-culture inserts	0/9	5/9	8/9	0/9	86%–93% (2)	4.3 cell layers (6)	++
Fibrin-coated culture plate	2/2	0/2	2/2	0/2		5–6 cell layers (2)	–
Fibrin gel	0/1	1/1	0/1	0/1	–	–	+++
Collagen IV-coated culture plate	1/1	0/3	0/1	1/1	–	4–12 cell layers (1)	+
Culture plate	0/4	4/4	3/4	0/4	–	2–6 cell layers (1)	+++

Number of studies using different culture parameters is presented in the Table; –, indicates not reported; +, low expression of p63; ++, moderate expression of p63; +++, high expression of p63.
